# Impact of Superparamagnetic Iron Oxide Nanoparticles on THP-1 Monocytes and Monocyte-Derived Macrophages

**DOI:** 10.3389/fmolb.2022.811116

**Published:** 2022-02-04

**Authors:** Christina Polasky, Tim Studt, Ann-Kathrin Steuer, Kristin Loyal, Kerstin Lüdtke-Buzug, Karl-Ludwig Bruchhage, Ralph Pries

**Affiliations:** ^1^ Department of Otorhinolaryngology, University Hospital of Schleswig-Holstein, Luebeck, Germany; ^2^ Institute of Medical Engineering, University of Luebeck, Luebeck, Germany; ^3^ Fraunhofer Research Institution for Individualized and Cell-Based Medical Engineering, Luebeck, Germany

**Keywords:** MPI, monocytes, iron oxide nanoparticles, biocompatibility, differentiation

## Abstract

Superparamagnetic iron oxide nanoparticles (SPIONs) are currently under examination for magnetic particle imaging, which represents a radiation free technology for three-dimensional imaging with high sensitivity, resolution and imaging speed. SPIONs are rapidly taken up by monocytes and other phagocytes which carry them to the site of inflammation. Therefore, the SPION biocompatibility is an essential parameter for a widespread MPI usage. Many improvements are expected from SPION development and its applications for cell visualization, but the impact of MPI optimized dextran coated SPIONs on the cellular characteristics of monocytic cells has been poorly studied up to now. THP-1 monocytes, monocyte-derived macrophages (MDM) as well as peripheral blood monocytes were incubated with MPI-optimized dextran-coated SPIONs of a size between 83.5 and 86 nm. SPION uptake was measured by FITC fluorescence of labeled SPIONs and Prussian blue staining. The activation of monocytes and MDMs was evaluated by CD14, CD11b and CD86 in flow cytometry. The secretion of IL-1β, and IL-10 was analyzed in supernatants. SPIONs were rapidly taken up by monocytes and monocyte-derived macrophages while no decrease in cell viability was observed. Expression patterns of CD11b, CD14, and CD86 were not affected in THP-1 monocytes and MDMs. Monocyte differentiation in macrophages was hindered during SPION uptake. THP-1 monocytes as well as monocyte-derived macrophages showed significantly increased IL-1β and decreased IL-10 secretion by tendency after SPION treatment. Dextran-coated SPIONs showed a low cytotoxicity on monocytes but exert undesirable inflammatory side effects that have to be considered for imaging applications.

## Introduction

In the past decade, nanoparticles (NPs) found their ways into the clinic as drug delivery systems and imaging contrast agents. There is a great variety of different organic materials such as liposomes, protein based and polymeric particles as well as inorganic metal NPs (Anselmo and Mitragotri 2016). Iron oxide (Fe3O4) NPs have been approved for iron deficiency and anemia treatments (Anselmo and Mitragotri 2019). Superparamagnetic iron oxide nanoparticles (SPIONs) additionally have been introduced in the clinic for numerous biomedical applications as nanocarriers in order to deliver therapeutic compounds towards targeted locations or for targeted thermal ablation of tumors (Quinto et al., 2015; Martinkova et al., 2018; Vangijzegem et al., 2019). The nanoscale structure as well as the features of superparamagnetism offer also a variety of possibilities for diagnosis. Due to their rapid uptake in phagocytes SPIONs are a very useful tool for imaging e.g., liver lesions, lymph node metastasis or atherosclerotic plaques (Anselmo and Mitragotri 2019; Dadfar et al., 2019). A novel very promising imaging modality is magnetic particle imaging (MPI), a technique that was first introduced by Gleich and Weizenecker in 2005 (Gleich and Weizenecker 2005). In contrast to MRI (magnetic resonance imaging) where similar particles are used, MPI provides a much lower detection threshold and significantly increased sensitivity and spatial resolution (Gleich and Weizenecker 2005; Panagiotopoulos et al., 2015). The nanoparticle design is an essential parameter for MPI measurements because the imaging principle is based on the superparamagnetic nature of SPIONs. Therefore, MPI resolution is depending on the SPION core homogeneity while the surface characteristics are key factors for biocompatibility and biosafety (Ai et al., 2011; Sanità et al., 2020). The MPI technique is ideally suited for applications such as vascular imaging and neuronal imaging as SPIONs are able to pass the blood-brain-barrier as well as cellular and targeted imaging (Weinstein et al., 2010; Panagiotopoulos et al., 2015).

Besides numerous advantages, the application of SPIONs to the human body involves the danger of cytotoxicity to the organism. The first reaction of the immune system to injected NPs is the uptake and elimination of the foreign particle (Geppert and Himly 2021). Monocytes are major phagocytes that accumulate these particles (Amani et al., 2021). Following internalization of dextran-coated SPIONs, the particles accumulate in the lysosome and the iron oxide is broken down into iron ions. Free iron can increase reactive oxygen species (ROS) production via Fenton and Haber-Weiss reactions resulting in oxidative stress, mitochondrial dysfunction, DNA damage and eventually inflammation (Wu et al., 2017; Patil et al., 2018; Geppert and Himly 2021). Furthermore, excess iron accumulates in the liver, spleen and kidney and can cause severe damage in these organs (Patil et al., 2018; Ghosh et al., 2020).

Nevertheless, monocytes are as sensitive part of the innate immune system crucial sensors for recognizing sites of inflammation and can therefore be used to detect those (Kirschbaum et al., 2016). For that reason, the loading of monocytes with MPI optimized SPIONs is a promising diagnostic tool for various inflammatory diseases. To evaluate the biocompatibility of MPI optimized SPIONs on human immune cells, we incubated THP-1 monocytes and primary monocytes with FITC-labeled dextran-coated SPIONs and measured activation parameters.

The aim of this study was to increase our understanding on immunological changes of human monocytes and monocyte derived macrophages (MDMs) in response to SPION uptake. In contrast to other SPIONs, the iron core diameter is very homogeneous with a mean hydrodynamic particle diameter between 83.5 and 86 nm, which is important for MPI measurements after phagocytosis by human cells (Lüdtke-Buzug et al., 2009a). The dextran coating has been described to facilitate SPION uptake cells decrease cytotoxicity (Balas et al., 2017; de Oliveira et al., 2017). FITC-labeling serves just as visualization tool to easily measure the cellular uptake by flow cytometry or fluorescence microscopy.

## Material and Methods

### Particle Preparation and Characterization

MPI optimized SPIONs were synthesized by the classical co-precipitation of iron oxide in an alkaline solution in the presence of dextran. For details on synthesis conditions and the purification processing chain see (Lüdtke-Buzug et al., 2009a; Lüdtke-Buzug et al., 2009b; Lüdtke-Buzug et al., 2013). The iron concentration was determined by photometry of the Prussian blue complex of the iron (Vámos and Gábor 1973). Through the several steps of the separation, the iron concentrations of the tracer solutions are between 0.22 and 0.25 mmol/ml (12–14 mg Fe). The hydrodynamic diameter (DH) has been determined by dynamic light scattering (Zetasizer Nano, Malvern) (Hendrix and Leipertz 1984), which allows for the simultaneous measurement of the particle size and the stability of the nanoparticles (Geers et al., 2007; Lüdtke-Buzug and Borchers 2012).

The stability of the particles has been analyzed by photon cross correlation spectroscopy (PCCS; Nanophox, Sympatec) before using them for the cell experiments. The hydrodynamic diameter of the SPIONs is stable in different media (demin. Water, PBS, different buffer) and at different temperatures (measured from 15 to 37°C) over different periods (minimum 21 days to 2 months) (Oberle and Lüdtke-Buzug, 2013; Katharina and Kerstin, 2015).

Magnetic properties of the particles have been analyzed by magnetic particle spectroscopy (MPS) before using them for the cell experiments. In MPS, the particles are subjected to a sinusoidally varying magnetic field. The magnetization response of the particles, however, is a non-linear periodic signal due to their super-paramagnetic properties. Since the non-linearity of the particle magnetization is connected with the iron-core diameter, this parameter can be estimated by solving the inverse problem based on the parameters of the Langevin theory (Biederer et al., 2009).

The particles have been produced by using Dextran, which is a biodegradable polymer. The Iron oxide is covalent connected to the Dextran and the particles are electrically neutral and potential can be measured (Zeta potential, Malvern).

Furthermore, we used the “*Genscript-ToxinSensor* ^*TM*^ Endotoxin Detection System” (GenScript Biotech, Leider, Netherlands) in order to measure possible endotoxin contaminations in the used SPION charges. This assay is based on an extract of blood cells from the “atlantic horseshoe crab” (*Limulus polyphemus*), which reacts with bacterial endotoxin lipopolysaccharide. *E. coli* endotoxin was used as an internal standard for the quantification of endotoxin in this chromogenic assay, which is measured at 545 nm.

To study the distribution of SPIONs in cells, the dextran was reacted with Isothiocyanatofluorescin in DMSO (dimethyl sulfoxide) under heating using DBTC (dibutyltin dichloride) as a catalyst. 1 g Dextran T70 was dissolved in 10 ml DMSO containing a few drops of pyridine. 0.1 g Isothiocyanatofluorescein was added, followed by 20 mg dibutyltin dilaurate, and the mixture was heated for 2 h at 95°C. After several precipitations in ethanol to remove free dye, the FITC-dextran was filtered off and dried in vacuum at 80°C (De Belder and Granath 1973).

### Monocytic Cell Line and Culture Conditions

For cell culture experiments and incubation with SPIONs the non-adherent monocyte cell line THP 1 was used. Cell culture was performed in RPMI 1640 medium supplemented with 10% heat inactivated fetal bovine serum (FBS), 1% sodium pyruvate and 1% streptomycin/penicillin at 37°C and 5% CO_2_ under a humidified atmosphere. Cells were subcultured every 3 days when they reached a maximum density of 1 × 10^6^ cells/ml.

THP-1 cells were incubated with SPIONs at concentrations of 20 and 50 µM Fe for different time points. To investigate time- and concentration-dependent SPION uptake, cell cultures were probed after 10, 20, 30, 60, 90, 180, 300, and 1,440 min of SPION incubation. Phagocytosis was determined by measuring FITC fluorescence by flow cytometry.

To induce differentiation into monocyte-derived macrophages (MDMs), THP-1 cells were treated with 10 ng/ml phorbol-12-myristate-13-acetate (PMA) for 24 h and washed once with fresh medium. After a two-day resting period, cells became adherent and developed a macrophage like morphology. Cells were either treated with SPIONs alone, SPIONs and PMA at the same time or first differentiated into MDMs and thereafter incubated with SPIONs for 24 h.

### SPION Incubation of Citrated Whole Blood

For incubation of primary peripheral monocytes, citrated whole blood from healthy volunteers (*n* = 3) was used. Healthy donors have given their written informed consent according to the approval of the local ethics committee of the University of Luebeck (approval number 18-332). Whole blood samples were diluted 1:2 in RPMI 1640 medium containing 10% heat inactivated fetal bovine serum (FBS), 1% sodium pyruvate and 1% streptomycin/penicillin. Incubation was likewise performed at 37°C and 5% CO2 under a humidified atmosphere for 6 h. SPION concentrations of 20 and 50 µM Fe were used.

### FACS Analysis

SPION incubated THP-1 cells were always washed twice in PBS before FACS analysis. For antibody staining, non-adherent THP-1 cells or trypsinized MDMs were harvested from cultures and centrifuged at 400 x g for 5 min. Cell pellets were incubated for 15 min in 5% BSA/PBS for Fc-Receptor blocking. Afterwards, cells were pelleted again and stained with the following antibodies at a dilution of 1:100 in PBS: CD14-APC-Cy7, CD11b-PerCP and CD86-BV421 (all from Biolegend, San Diego, United States). Additionally, Propidium iodide (PI) staining was performed to determine cellular viability. 2 µL PI solution (BD, Franklin Lakes, NJ, United States) per tube were added to the antibody cocktail. After 20 min of staining in the dark, cells were washed twice and the pellets were resuspended in 0.5 ml PBS.

Flow cytometry was performed with a MACSQuant 10 flow cytometer (Miltenyi Biotec, Bergisch-Gladbach, Germany) and data were analyzed using the FlowJo software version 10.0 (FlowJo, LLC, Ashland, United States).

The incubated whole blood was stained with CD45-PE, CD16-APC, CD14-APC-Cy7 and HLA-DR-BV421 (1:100; Biolegend) for 20 min in the dark. Red blood cell lysis was performed by adding 700 µL RBC-Lysis Buffer (Biolegend) for another 20 min. Subsequently, suspension was centrifuged at 400 x g for 5 min and supernatant was discarded. 100,000 CD45^+^ leukocytes were analyzed. Gating of monocyte subsets was performed as described before (Polasky et al., 2021). All antibody titrations and compensations were performed in beforehand.

### IF/ICC

THP-1 cells were incubated for 24 h with SPIONs at a concentration of 20 and 50 µM Fe. Afterwards, a cell suspension of not more than 0.5 × 10^6^ cells/ml was prepared in 5% BSA/PBS. For cytospins 200 µL of cell suspension/slide were centrifuged at 800 rpm for 5 min (Thermo Scientific™ Cytospin™ 4 cytocentrifuge, Fisher Scientific GmbH, Schwerte, Germany). Slides were dried at room temperature for 1 h, then fixated for 10 min in ice cold acetone and frozen until use.

For Immunofluorescence slides were rehydrated in PBS and incubated with an autofluorescence quenching kit (Vector^®^ TrueVIEW^®^ Autofluorescence Quenching Kit, Burlingame, California, United States) according to standard protocol. Slides were coverslipped with watery mounting medium containing 4,6-diamidino-2-phenylindole (DAPI, 1 μg/ml; Roche Diagnostics, Mannheim, Germany) for nuclei staining.

For Immunocytochemistry we performed Fe-III-ions detection with Perls’ Prussian blue. Cytospins were rehydrated with PBS and then immersed in a solution of equally mixed 20% hydrochloric acid and 10% potassium ferrocyanide for 45 min at 37°C. After washing three times with distilled water, counterstaining with nuclear fast red (Carl Roth GmbH + Co. KG, Karlsruhe, Germany) for 7 min was performed. Slides were rinsed twice in distilled water and coverslipped with watery mounting medium. All slides were photographed using an optical microscope (AxioVision, Zeiss, Oberkochen, Germany).

### Cytokine Analysis

To determine monocytic cytokine expression patterns in responses to SPION uptake, enzyme-linked immunosorbent assay (ELISA) was performed. Supernatants from cell cultures were collected after 24 h SPION incubation of monocytes or from MDM incubated with SPIONs and instantly frozen with liquid nitrogen and preserved at −80°C. The protein concentrations of the human IL-1β and IL-10 were determined according to the protocol given by the commercial ELISA kits (R&D Systems, United States).

### Statistical Analysis

Statistical analyses were performed with GraphPad Prism Version 7.0f. The mean and standard error (SEM) are presented. The differences between groups were determined after testing for Gaussian distribution (normality tests), and applying parametric (student`s *t*-Test), or non-parametric 1-way Anova with Bonferroni post hoc test. The correlation between parameters was calculated using multivariate regression with the Pearson correlation coefficient. *p* < 0.05 (*), *p* < 0.01 (**), *p* < 0.001 (***), and *p* < 0.0001 (****). Additional statistical details are given in the respective figure legends, when appropriate.

## Results

### Characteristics of Used SPIONs

**FIGURE 1:**
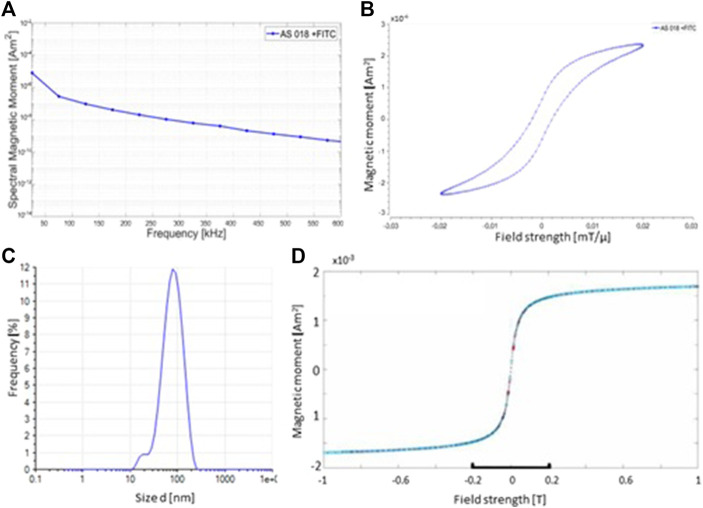
Physical characteristics of synthesized SPIONs. **(A)** MPS measurement of the SPINOs shows the amplitude spectrum of the odd harmonic. The amplitude spectrum of the FITC-labeled nanoparticles indicates a good signal response for imaging by MPI. **(B)** MPS measurements illustrate the magnetization curve of the SPIONs. **(C)** Photon correlation spectroscopy (PCS) measurements of the size distribution of the nanoparticles. The mean hydrodynamic diameter of the used SPIONs is 67.0 nm **(D)** The magnetic properties of the particles were verified using a vibrating sample magnetometer (VSM) at room temperature.

The physical characteristics of the used superparamagnetic nanoparticles were evaluated by varoious methods. Figure 1A shows the amplitude spectrum of the FITC-labeled particles. In the semi-logarithmic amplitude spectrum of the particle magnetization, it can be seen that the height of the amplitudes falls approximately linearly with the frequency decrease. The slope of this decrease can provide information as to whether particles are suitable for MPI. This is because the larger the slope of a compensation line, the better the quality of the particles for imaging. The graph exhibits such a linearly decreasing slope. This indicates a good signal response of the particles for imaging by MPI.

In addition, an endotoxin detection assay was used in order to measure possible endotoxin contaminations in the synthesized SPION charges. No endotoxin was detectable in all measured SPION probes.

The behavior of the magnetic moments of a SPION after application of an external magnetic field results in a magnetization curve in the form of a hysteresis loop. If the diameter of a ferromagnetic particle falls below a critical size (approx. 100 nm), it consists of only one Weiss district. The magnetization curve then no longer runs in a hysteresis loop as in a ferromagnetic material, but sigmoidally (as in a paramagnetic material). A ferromagnetic substance which behaves paramagnetically on a macroscopic level is called superparamagnetic. The magnetization curve of the nanoparticles shows almost a sigmoidal shape. The difference in magnetic field strength from the origin that can be seen is a deviation from the ideal value to the real value (Figure 1B). Since this deviation is less than 0.005 mT/µ, it is such a small divergence that it can be neglected here. Essential for the magnetic behavior of the particles is the iron-containing core. The size of this core can also be determined using the MPS. The SPIONs used for this work have an average core diameter of 6.48 nm.

Furthermore, the hydrodynamic diameter of the SPIONs was measured by Photon Correlation Spectroscopy (PCS). Compared to the core diameter, which is crucial for the magnetic behavior of the nanoparticles, the hydrodynamic diameter is an important property for the behavior of the SPIONs in tissue/cells. Depending on the target site, the hydrodynamic diameter must not exceed certain sizes, otherwise they cannot be taken up by the desired tissue or cells. The ideal hydrodynamic diameter of the particles for this series of experiments is between 60 and 80 nm. From the PCS measurement shown, the average hydrodynamic diameter of the particles used is 67.00 nm Figure 1C). The hydrodynamic diameter is measured using a photon correlation spectrometer (PCS). The measurement method is based on the principle of dynamic light scattering, in which the scattered light from a laser on the particle suspension indicates the velocity of the particles and the hydrodynamic diameter can be calculated using the Stokes-Einstein relationship.

### Time and Concentration Dependent SPION Uptake by THP-1 Monocytes

**FIGURE 2:**
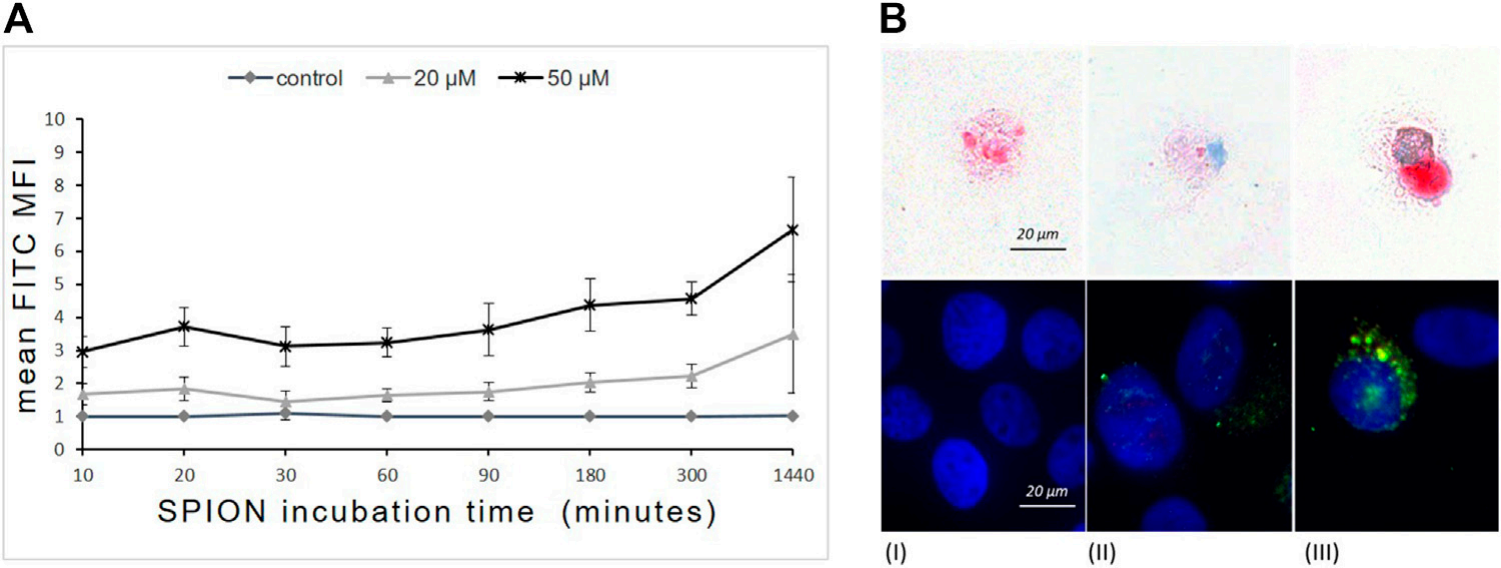
Time- and concentration-dependent uptake of dextran-coated SPIONs by THP-1 monocytes. **(A)** FITC fluorescence (mean fluorescence intensity = MFI) of THP-1 cells measured by FACS that represents SPION uptake over time. *n* = 6. **(B)** Prussian`s blue staining of Fe (upper panel) and immunofluorescence (lower panel) of FITC-labeled SPIONs in THP-1 monocytes after 24 h of incubation. (I) control; (II) 20 µM Fe; (III) 50 µM Fe. Red arrows indicate iron accumulations in the cells. Blue fluorescence shows DAPI stained nuclei and green indicates FITC-labeled SPIONs.

To evaluate the dynamic of SPION phagocytosis by human monocytes, THP-1 cells were incubated with SPIONs and samples were analyzed after 10 min up to 1,440 min (24 h). SPION uptake was measured by flow cytometry and fluorescence microscopy as SPIONs were visualized based on their fluorescent FITC-labeling. In addition, SPIONs were detected using Perls’ Prussian blue staining of iron ions (Figure 2). THP-1 cells without SPIONs were used as internal control.

Flow cytometry revealed a rapid concentration-dependent cellular uptake of SPIONs starting already after 10 min of incubation. The phagocytosis of SPIONs detected by FITC fluorescence of monocytes increased steadily at both concentrations over the first 300 min and proceeded until 1,440 min (24 h) (Figure 2A). The FITC fluorescence was significantly higher than in controls at any time point.

Microscopic investigations revealed a cytoplasmic localization of SPIONs (Figure 1B). Furthermore, THP-1 cells showed concentration dependent differences in the amount of phagocyted SPIONs (Figure 2B).

### THP-1 Phenotype During SPION and PMA Incubation

**FIGURE 3:**
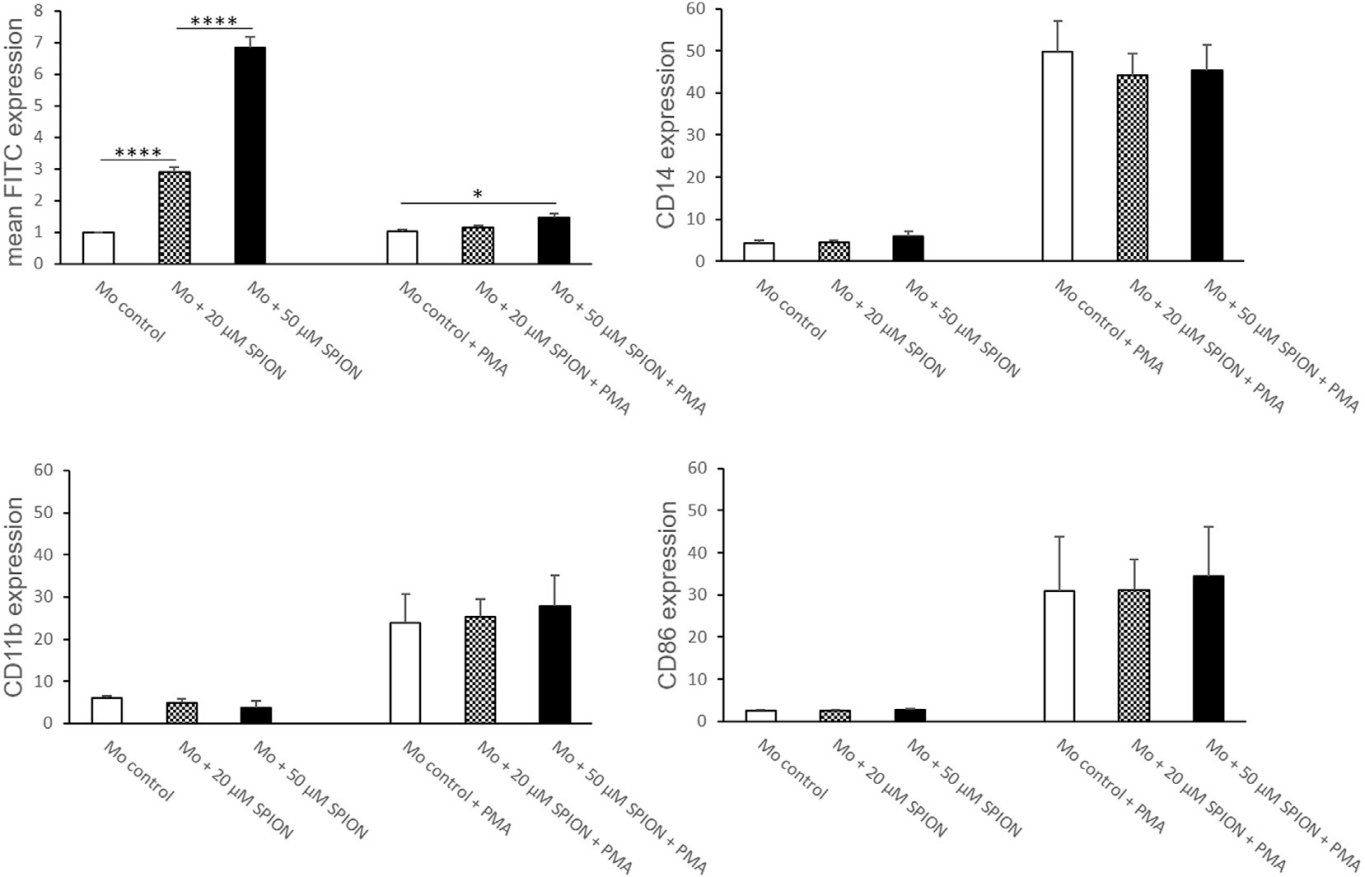
Phenotype of SPION incubated THP-1 cells with and without additional PMA treatment for macrophage differentiation. Cells were either incubated with SPIONs alone or in combination with PMA to induce macrophage differentiation. SPION incubation was performed over 24 h. Cells without SPIONs served as control. FITC expression of cells represents SPION uptake. Shown are the mean fluorescence intensities (MFI) of each measured protein on the cell surface. Significance: student`s *t*-Test. *p ≤ 0.05; ****p ≤ 0.0001. *n* = 6.

THP-1 cells were co-cultured with 20 or 50 µM SPIONs. SPION uptake as well as cell viability and expression of monocyte specific markers were evaluated after 24 h. FITC fluorescence of monocytes was significantly increased in a concentration dependent manner (Figure 3). Cell viability was not influenced by SPIONs. The expression of CD14 and CD86 was not altered by SPION uptake (Figure 3).

When THP-1 cells were additionally stimulated with PMA, SPION uptake was strongly inhibited compared to cells without PMA, but was still increased in 50 µM Fe cultures compared to controls (Figure 3). The cell viability was overall lower in PMA-stimulated cultures, but likewise not influenced by SPIONs. The expression of CD14 and the macrophage-specific marker CD86 was strongly and significantly increased due to PMA treatment compared to cultures without PMA. However, SPION incubation did not alter expression of these markers (Figure 3).

### THP-1 Monocyte Derived Macrophage Phenotype After SPION Phagocytosis

**FIGURE 4:**
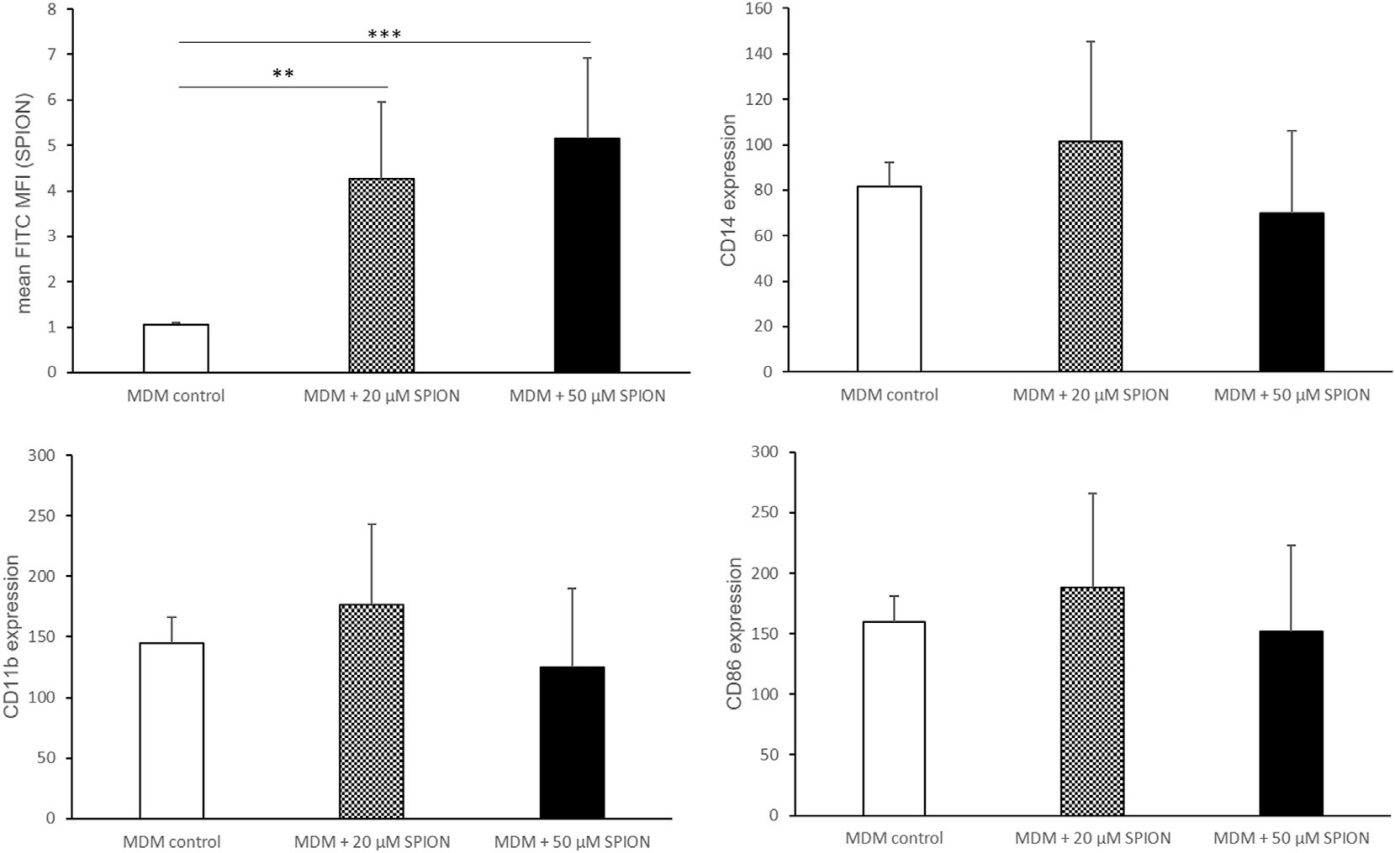
Phenotype of SPION incubated monocyte-derived macrophages (MDMs). PMA-stimulated MDMs were incubated with or without SPIONs for 24 h. FITC expression of cells represents SPION uptake. Shown are the mean fluorescence intensities (MFI) of each measured protein on the cell surface. Significance: student`s *t*-Test. **p ≤ 0.01; ***p ≤ 0.001. *n* = 6.

PMA stimulation for 24 h followed by a resting period of 48 h induced a differentiation of THP-1 monocytes into monocyte-derived macrophages (MDMs). An incubation with SPIONs thereafter led to a significant increase of FITC fluorescence of the cells that represents SPION phagocytosis. The monocyte specific cell surface proteins CD11b and CD14 were not altered by SPION incubation. In addition, the expression of the macrophage specific marker CD86 was strongly increased on MDMs compared to naïve THP-1 monocytic cells as well as not fully differentiated MDMs, but was also not altered due to SPION incubation of the cells (Figures 3, 4).

### Cytokine Production of THP-1 Cells in Response to SPION Uptake and PMA Treatment

As additional functional parameter we investigated whether SPION uptake could modulate the production of immunoregulatory cytokines interleukin (IL)-1ß and IL-10 by THP-1 monocytes and monocyte derived macrophages *in-vitro*. Cytokines were analyzed in response to SPION phagocytosis and PMA induced differentiation. Cell culture supernatants from monocytic THP-1 cells as well as PMA induced MDMs were analyzed.

**FIGURE 5:**
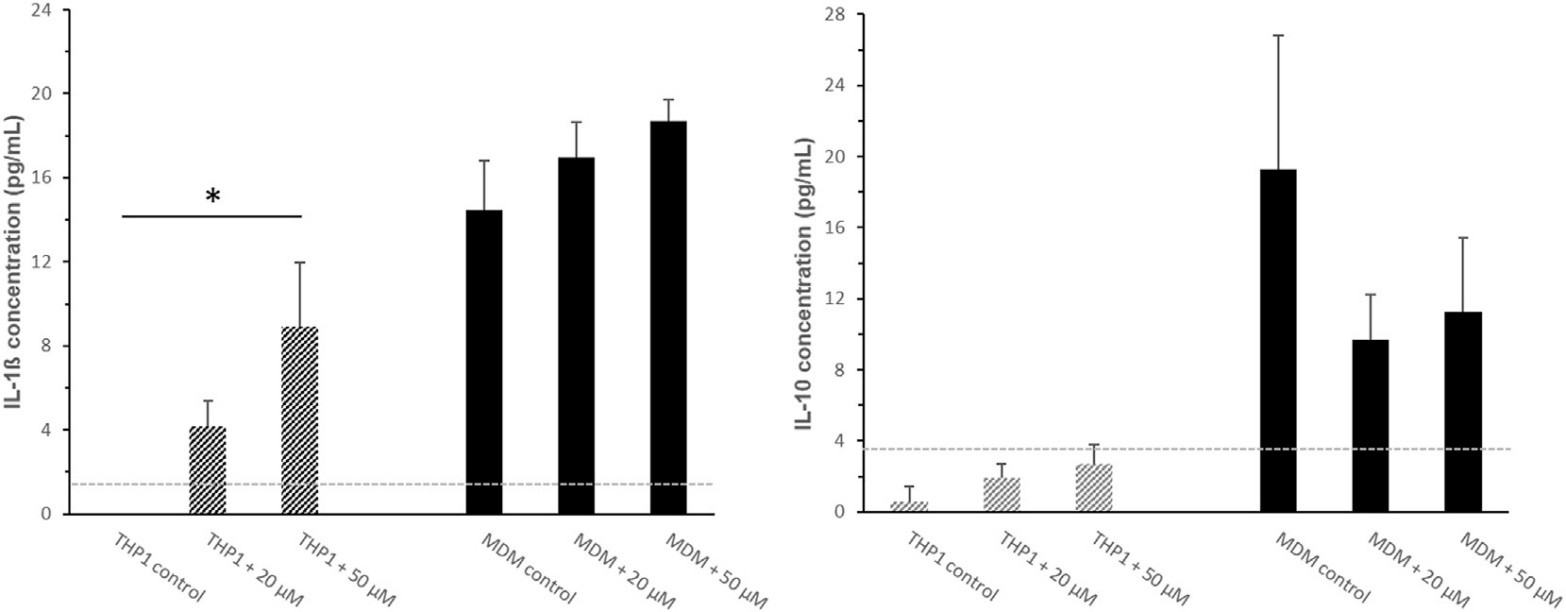
Cytokine secretion of monocytic THP-1 cells and monocyte-derived macrophages (MDMs) after 24 h SPION incubation. The pro-inflammatory cytokine IL-1β and the anti-inflammatory cytokine IL-10 were analyzed in cell culture supernatants by standard ELISA protocol. The light grey dotted line represents the detection limit. Significance: student`s *t*-Test. *p ≤ 0.05. *n* = 3.

THP-1 cells alone showed no secretion of the pro-inflammatory cytokine IL-1β. Incubation with SPIONs however led to a significant increase of the IL-1β release from the cells (Figure 5). PMA-induced MDMs showed a significantly increased secretion of IL-1β compared to monocytic THP-1 that was also even more increased in SPION incubated cultures by tendency. The level of the anti-inflammatory cytokine IL-10 was very low in monocytic THP-1 cultures. A differentiation towards macrophages led to a significantly increased secretion of IL-10. MDM cultures that were incubated with SPIONs revealed a lower IL-10 level compared to controls by tendency (Figure 5).

### SPION Uptake by Primary Peripheral Blood Monocytes

**FIGURE 6:**
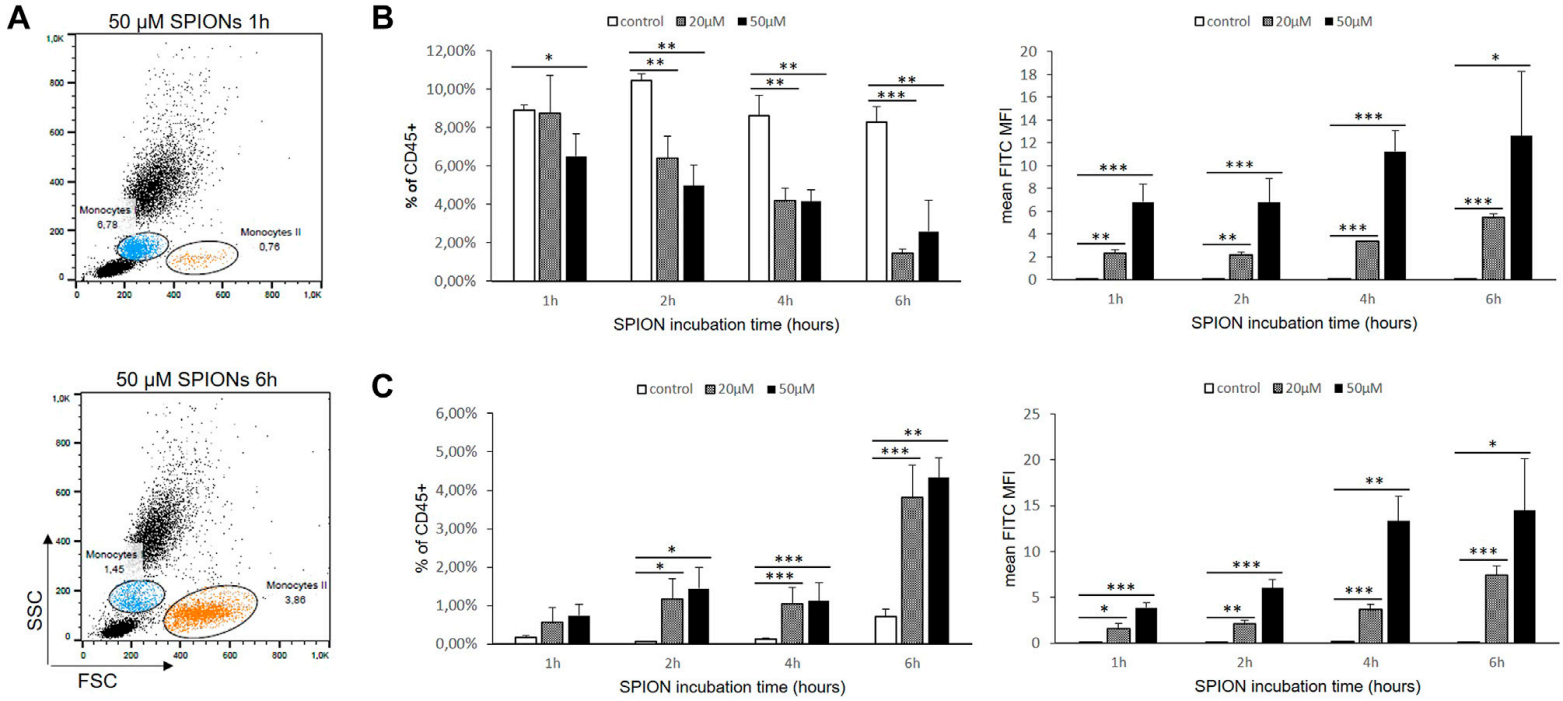
SPION uptake by primary monocytes in whole blood. **(A)** Dot Blots of FACS analyses from one representative sample after 1 and 6 h SPION incubation. Regular monocytes (Monocytes I) are colored in blue and larger-sized monocytes (Monocytes II) are colored in orange. **(B)** Percentage of Monocytes I and their FITC fluorescence (MFI) in SPION-incubated cultures over time. **(C)** Percentage of Monocytes II and their FITC fluorescence (MFI) in SPION-incubated cultures over time. Significance: student`s *t*-Test. *p ≤ 0.05; **p ≤ 0.01; ***p ≤ 0.001. *n* = 3.

Human whole blood was diluted 1:2 in RPMI 1640 medium and incubated at 37°C with or without SPIONs. Monocytes were gated according to their FSC/SSC characteristics and expressed as percentages of measured total CD45^+^ leukocytes. The monocyte amount decreased significantly under SPION incubation and over time. In contrast, another putative monocyte population developed in the cultures starting after 1 h (Monocytes II; Figure 6A). This population was characterized by higher FSC values representing a greater cell size. The percentages of this larger-sized population were significantly increased in both SPION incubated cultures starting after 2 h (Figures 6B,C). Both monocyte populations showed in SPION-incubated blood a significantly increased FITC fluorescence representing SPION uptake starting after 1 h and progressing until 6 h (Figures 6B,C). The expression of monocyte specific markers as well as the CD14/CD16 classified subsets could not be analyzed anymore in whole blood cultures.

## Discussion

Aim of the present study was to evaluate the biocompatibility of superparamagnetic SPIONs and monocytes for future magnetic particle imaging to find inflammatory sites. Furthermore, the questions of a possible immunoregulatory impact of SPIONs on monocytes was addressed for THP-1 monocytes and primary human monocytes.

In general, the influence of NPs on cells depends on various factors such as size, shape, surface charge and coating of the used particles as well as bystander substances (Shah and Dobrovolskaia 2018; Geppert and Himly 2021). Most critical issue of using nanoparticles is their cytotoxicity. The exposure of SPIONs to cells has been associated with significant toxic effects such as inflammation, formation of apoptotic bodies, impaired mitochondrial function, membrane leakage and DNA damage (Singh et al., 2010; Ghosh et al., 2020). However, data remains somewhat controversial as current literature agrees that iron oxide nanoparticles are most secure and non-cytotoxic at concentrations below 100 mg/ml (Jeng and Swanson 2006; Singh et al., 2010). There is also a lot of evidence that dextran-coating of iron-oxide nanoparticles not only improves the active uptake by cells, but also decreases their cytotoxic side effects (Yu et al., 2012; Balas et al., 2017; de Oliveira et al., 2017). Data from our study corroborates prior findings that the uptake of our dextran-coated SPIONs occurred very quickly and the cell viability of THP-1 monocytes was not altered upon SPION administration.

Nevertheless, SPIONs have cytotoxic capabilities, first of all by causing oxidative stress. It has been repeatedly shown that SPION uptake leads to activation of monocytes resulting in an increased production of ROS and pro-inflammatory cytokines such as IL-1β, IL-6, and TNF-α (Zhu et al., 2011; Escamilla-Rivera et al., 2015; Strehl et al., 2015; Gonnissen et al., 2016). In order to investigate potential M1/M2 polarizing or inflammatory effects of SPIONs, we analyzed different marker proteins such as CD11b, CD14 and CD86 as well as the secretion of immune regulatory cytokines IL-1ß and IL-10. CD14 represents a cell surface protein involved in immune responses against bacterial lipopolysaccharides. In association with TLR4, CD14 triggers the secretion of pro-inflammatory cytokines (Zamani et al., 2013). It has recently been reported that SPIONs may be able to induce TLR4 signaling cascades (Jin et al., 2019). Mulens-Arias and colleagues showed a TLR4-mediated activation of monocytes by SPIONs that modulated ROS production and podosome formation (Mulens-Arias et al., 2015). CD11b represents integrin-α M, a cell molecule important for regulating cell adhesion, phagocytosis and chemotaxis. The macrophage specific marker CD86 was analyzed to check for macrophage differentiation patterns of monocytes. Our results show that SPION uptake alone did not induce macrophage differentiation of THP-1 monocytes. Additionally, CD14 and CD11b expression was not affected by SPION uptake. The CD14 and CD86 expression of THP-1 cells was only upregulated after PMA incubation and induction of MDM differentiation. Taken together, the expression of specific surface proteins on THP-1 monocytes was not altered by SPION incubation. The situation *in-vivo* could unfortunately not be studied as blood showed a lot of clumping and cell degradation. The relevant surface markers were not reliably measureable in whole blood cultures. The increase of bigger-sized monocytes that was significantly induced by SPIONs indicates a differentiation towards more macrophage-like cells or an inflammatory polarization of the monocytes. Another possibility is the mere swelling nearly bursting of the cells after the phagocytosis of the SPIONs. This would indicate a rather severe cytotoxic effect of the nanoparticles. These experiments have to be addressed in depth in future studies to exclude such dramatic effects.

The secretion of the pro-inflammatory M1 cytokine IL-1ß was clearly induced by SPIONs. A differentiation towards a pro-inflammatory phenotype was reinforced by the lowered IL-10 production in THP-1 MDMs after SPION administration. Monocytes are the major source of IL-1ß in response to inflammation. On the other side, macrophages are known to secrete immunoregulatory cytokine IL-10 in response to phagocytosis of apoptotic cells in order to ensure cellular homeostasis. In contrast to recent publications, dextran-coating of our SPIONs could not completely abolish unwanted pro-inflammatory side effects on THP-1 monocytes. A SPION-induced activation of monocytes as well as a phenotypic shift of M2-like towards M1-like macrophages have been repeatedly proposed (Laskar et al., 2013; Rojas et al., 2016). Another publication proved an activation of MAPK signaling pathways in primary human monocytes that resulted in secretion of pro-inflammatory cytokines (Wu et al., 2018). Sindrilaru and colleagues even suggested an iron-mediated regulation of the functional plasticity of monocytes (Sindrilaru et al., 2011). These findings may point to dramatic side effects relating to the involvement of iron-activated M1 macrophages in endothelial damage and the formation of atherosclerotic plaques (Zhu et al., 2011; Laskar et al., 2013). In contrast, the immunomodulatory mechanism of SPIONs triggers the macrophage polarization in tumor tissue towards a M1-like anti-tumor phenotype (Reichel et al., 2019; Mulens-Arias et al., 2021). These phenomenon is a promising approach for tumor therapy and has been approved in cell culture and murine models (Poller et al., 2017; Zhang et al., 2020). Other publications could not prove an immunoregulatory impact of iron oxide nanoparticles of the size of 10 and 30 nm on the production of cytokines as tumor necrosis factor-α, IL-6, and IL-1β or altered endothelial interaction of primary human monocytes (Matuszak et al., 2015; Grosse et al., 2016).

The kind of metal as well as the iron core size seems to be highly important in terms of biocompatibility (Jeng and Swanson 2006). These contradictory results point out the impact of particle size and coating of SPIONs on immune cell reactions. Magnetic resonance imaging (MRI) is a very well established tool using superparamagnetic iron oxide nanoparticles (SPIONs) to track immune and stem cells used for cellular therapies. Although SPION-based MRI cell tracking has a high sensitivity for cell detection, it is unsatisfactory in order to quantify SPION tissue concentrations or reliable cell numbers, respectively (Bulte et al., 2011).

Magnetic particle imaging (MPI) was introduced in 2005 as a novel imaging modality and provides great potential for overcoming these deficiencies of MRI-based cell tracking (Gleich and Weizenecker, 2005). In early MPI cell analyzes, commercially available SPIONs as used for MRI, such as ferucarbotran, have been used in order to detect neural progenitor cells or T-lymphocytes (Zheng et al., 2015; Rivera-Rodriguez et al., 2020). In the meantime, ferucarbotran is not considered for optimal MPI cell tracking anymore because its limitations with regards to size distribution and the formation of aggregates (Eberbeck et al., 2013). Therefore, we investigated the influence of MPI optimized dextran coated SPIONs on THP-1 and primary monocytes as well as monocyte derived macrophages with respect to different immunological aspects such as viability, expression of activation markers or cytokine secretion, respectively.

Nevertheless, there are still different aspects of this study that require further investigations. *In vivo* distribution and immune functions of the different monocyte subsets are known to react sensitively to different kinds of environmental alterations such as injuries or inflammatory disease (Patel at al., 2017). Therefore, the repertoire of cellular parameters which must be taken into account is correspondingly complex, only a few of which could have been analyzed in this work.

Data from our study confirmed recent findings that dextran-coated SPIONs are rapidly taken up and exert only low toxicity on monocytes. However, there was a clear induction of the expression of the pro-inflammatory cytokine IL-1β which indicates unwanted inflammatory side effects. The impact on primary monocytes remains elusive. Furthermore, the study concentrated on THP-1 cells as a immunological model system and included only a limited sample size, which is a clear limitation in regard to transferability of the data to human applications. The quality requirements of SPIONs in order to use them as imaging tool have to be carefully considered and need to be studied further in detail.

## Data Availability

The original contributions presented in the study are included in the article/supplementary material, further inquiries can be directed to the corresponding author.
